# Functional role of long-lived flowers in preventing pollen limitation in a high elevation outcrossing species

**DOI:** 10.1093/aobpla/plx050

**Published:** 2017-10-21

**Authors:** Mary T K Arroyo, Diego Andrés Pacheco, Leah S Dudley

**Affiliations:** Instituto de Ecología y Biodiversidad, Casilla 653, Santiago, Chile; Departamento de Ciencias Ecológicas, Facultad de Ciencias, Universidad de Chile, Casilla 653, Santiago, Chile; Biology Department, University of Wisconsin-Stout, Menomonie, WI 54751

**Keywords:** Alpine, Amaryllidaceae, Andes, floral senescence, flower longevity, flower visitation rates, global warming, low-quality pollen, pollen limitation, *Rhodolirium montanum*

## Abstract

Low pollinator visitation in harsh environments may lead to pollen limitation which can threaten population persistence. Consequently, avoidance of pollen limitation is expected in outcrossing species subjected to habitually low pollinator service. The elevational decline in visitation rates on many high mountains provides an outstanding opportunity for addressing this question. According to a recent meta-analysis, levels of pollen limitation in alpine and lowland species do not differ. If parallel trends are manifested among populations of alpine species with wide elevational ranges, how do their uppermost populations contend with lower visitation? We investigated visitation rates and pollen limitation in high Andean *Rhodolirium montanum*. We test the hypothesis that lower visitation rates at high elevations are compensated for by the possession of long-lived flowers. Visitation rates decreased markedly over elevation as temperature decreased. Pollen limitation was absent at the low elevation site but did occur at the high elevation site. While initiation of stigmatic pollen deposition at high elevations was not delayed, rates of pollen arrival were lower, and cessation of pollination, as reflected by realized flower longevity, occurred later in the flower lifespan. Comparison of the elevational visitation decline and levels of pollen limitation indicates that flower longevity partially compensates for the lower visitation rates at high elevation. The functional role of flower longevity, however, was strongly masked by qualitative pollen limitation arising from higher abortion levels attributable to transference of genetically low-quality pollen in large clones. Stronger clonal growth at high elevations could counterbalance the negative fitness consequences of residual pollen limitation due to low visitation rates and/or difficult establishment under colder conditions. Visitation rates on the lower part of the elevational range greatly exceeded community rates recorded several decades ago when the planet was cooler. Current pollen limitation for some species in some habitats might underestimate historical levels.

## Teaser

We asked whether an expected elevation reduction in flower visitation rates is compensated by long-lived flowers in the high Andean geophyte, Rhodolirium montanum. Although partial flower longevity compensation was found, the functional role of R. montanum’s long-lived flowers was strongly masked by a second source of pollen limitation attributable to a greater tendency for geitonogamous pollination on larger clones at higher elevations. The long-term fitness benefits of clonal growth in the harsh alpine environment appear to outweigh the seasonal fitness losses associated with residual quantitative pollen limitation plus additional qualitative pollen limitation.

## Introduction

A plant is said to be pollen limited when its fruit or seed set falls below the maximum a plant′s resources can sustain as a result of insufficient availability of pollen (Burd 1994; Larson and Barrett 2000; Ashman *et al.* 2004; Knight *et al.* 2005). Insufficient pollen can result from pollinator fluctuation driven by climatic factors such as inclement weather (Arroyo *et al.* 1985; Corbet 1990; McCall and Primack 1992; Totland 1994; Torres-Díaz *et al.* 2007; Tuell and Isaacs 2010; Fulkerson *et al.* 2012). Competition for pollinators among co-flowering species (Bell *et al.* 2005; Vamosi *et al.* 2013), inefficient pollen transfer by low-quality pollinators (Gómez *et al.* 2007) and heterospecific pollen transfer (Morales and Traveset 2008; Tur *et al.* 2016) are other sources of pollen deficits. Deposition of genetically low-quality pollen on stigmas can lead to qualitative as opposed to quantitative pollen limitation (Aizen and Harder 2007; Harder and Aizen 2010; Arceo-Gómez and Ashman 2014). This source of pollen limitation is more difficult to detect but can be significant. In addition to these various extrinsic factors, absolute levels of pollen limitation are shaped by breeding system (Larson and Barrett 2000; Knight *et al.* 2005; Vamosi *et al.* 2013), ovule bet-hedging (Burd 1995; but see Rosenheim *et al.* 2016), and parental optimism (Rosenheim *et al.* 2014). Persistent pollen limitation can threaten population persistence (e.g. Robertson *et al.* 1999; Pattemore and Anderson 2013). Consequently, in outcrossing species habitually subject to low pollen availability, we expect to find functional traits or adaptations leading to the avoidance of strong pollen limitation.

In the alpine zone above the treeline, where air temperature descends at an average global rate of 6.0 K per 1000 m increase in elevation (Körner 2003), flower-visitation rates are known to decline with increasing elevation (Arroyo et al. 1982, 1985; Arroyo and Squeo 1990; Bingham and Orthner 1998). However, while many alpine species show some pollen limitation (e.g. Galen 1985; Elberling 2001; Totland and Sottocornola 2001; Kasagi and Kudo 2003; Muñoz and Arroyo 2006; Torres-Díaz *et al.* 2011; Ai *et al.* 2013; Lázaro *et al.* 2015), a meta-analysis found no difference in the intensity of pollen limitation between alpine and lowland species (García-Camacho and Totland 2009; see also Lázaro *et al.* 2015). This result suggests that an analogous trend could be expected among populations of outcrossing alpine species situated at different elevations above the treeline. That is, if alpine and lowland species, in general, do not differ in pollen limitation, individual populations of alpine species found along the elevational gradient above the treeline are not expected to either in spite of demonstrated decreases in visitation rates from the treeline upwards. Under these circumstances we expect alpine species to possess traits that enable their high elevation populations to overcome pollen limitation. Understanding how outcrossing species avoid pollen limitation in the alpine could shed light on how species in other habitats characterized by harsh conditions for pollinators (e.g. Arctic, tropical cloud forests) deal with this problem.

High elevation populations of alpine species could avoid excessive pollen limitation via the possession of long-lived flowers (Arroyo et al. 1981, 1985, 2006; Primack 1985; Bingham and Orthner 1998; Steinacher and Wagner 2010; Torres-Díaz *et al.* 2011). This hypothesis, coined the ‘increased pollination probability hypothesis’ (Arroyo *et al.* 2006), sees long-lived flowers allowing a plant to ‘sit and wait’ for greater amounts of time to be visited when pollinators are scarce (cf. Ashman *et al.* 2004). Long-lived flowers are an intrinsic property of some alpine species (Steinacher and Wagner 2010; Torres-Díaz *et al.* 2011; Pacheco *et al.* 2016). Although potential flower longevity (measured in pollinator-excluded flowers) does not necessarily increase with elevation in such species, compensation of lower visitation rates at higher elevations can be expected. High seed set in some high nival plants with fairly long-lived flowers is associated with pollen deposition over several days of the flower life span (Wagner *et al.* 2016). In some alpine species, potential flower longevity increases with elevation (Utelli and Roy 2000; Blionis and Vokou 2001; Giblin 2005; Duan *et al.* 2007; Ai *et al.* 2013; Trunschke and Stöcklin 2016). Such increases could be the result of selection on flower longevity (Utelli and Roy 2000), but in many cases are likely to be the result of the plastic extension of the flower lifespan under cooler temperatures and which can compensate for slower pollination (cf. Arroyo *et al.* 2013).

Our goals were to compare elevational trends in flower-visitation rates and pollen limitation and to examine the functional role of a species’ intrinsically long-lived flowers in compensating for lower visitation rates at high elevations in the high Andes of central Chile. We used *Rhodolirium montanum* (Amaryllidaceae) as a focal species. Like many amaryllids (e.g. Arroyo and Dafni 1995; Baker *et al.* 2000), *R. montanum* is characterized by large long-lived flowers (Pacheco *et al.* 2016). If flower longevity compensation occurs at higher elevations in *R. montanum*, levels of seed set should be greater than expected from the depression in visitation rates. Because we expect lower flower visitation rates at higher elevation, stigmatic pollen deposition should commence later in the flower lifespan and/or occur in smaller daily dosages. Under slower or less intense pollination, given that pollination provokes floral senescence (Lundemo and Totland 2007; Castro *et al.* 2008; Shahri and Tahir 2011; Arroyo *et al.* 2013), we expect realized flower longevity to become more prolonged with increasing elevation. The overall aims were to investigate whether pollen limitation remains constant over the elevational range of *R. montanum* as occurs at a large spatial scale for alpine versus lowland species, and to shed light upon the functional role of flower longevity in compensating for lower visitation rates at the higher elevations.

## Methods

### Study species and area

**Figure 1.**
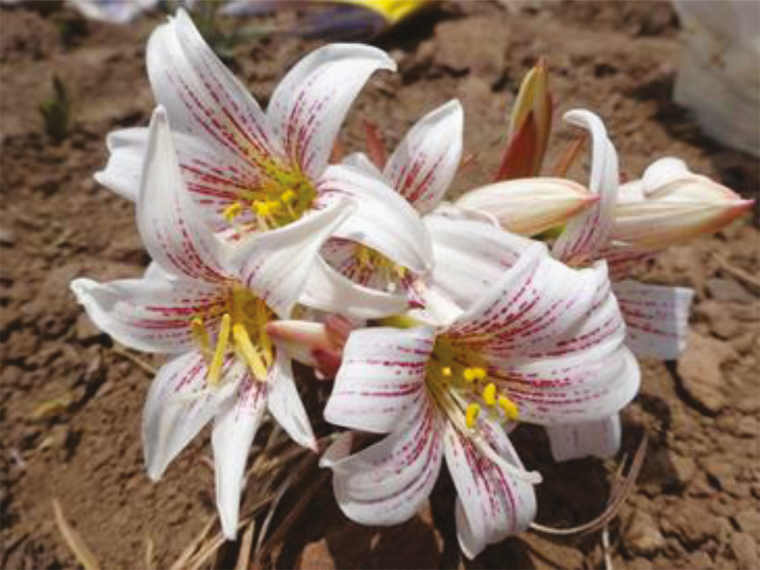
*Rhodolirium montanum* (Amaryllidaceae). Image: from Pacheco *et al.* (2016).

*Rhodolirium montanum* (syn: *Rhodophiala rhodolirion*) is a large-flowered, high elevation Andean geophyte (Fig. 1). In the Farellones-Valle Nevado area (33° Latitude S) where the present work took place, *R. montanum* is distributed over 1400 m of elevation (1800–3200 m a.s.l.). *R. montanum* becomes increasingly clonal with elevation. Single-bulbed individuals are commonly found on the lower end of the elevational range but become increasingly rare as elevation increases. Genets comprising up to 21 loosely connected flowering bulbs have been found in the upper part of the elevational range (D. Pacheco, unpubl. data). Each large bulb produces a robust peduncle supporting a single (rarely two) nectarless flowers. *R. montanum* is partially self-compatible, strongly herkogamous and mostly outcrossing (Ladd and Arroyo 2009; Pacheco *et al.* 2016). Elevational tendencies in potential flower longevity measured in pollinator-excluded flowers have been extensively studied by Pacheco *et al.* (2016). In more normal years, mean potential flower longevity stands at 8.1 days and does not vary with elevation. Some individual flowers have been reported to remain open up to 16 days. However, in dry years, by default, potential flower longevity becomes longer at the higher elevations as result of a plastic shortening of the flower lifespan due to intensive soil drying at lower elevation. Open-pollinated flowers have been reported to remain open 7.7 days (Ladd and Arroyo 2009). This exceeds the average realized flower longevity for non-asteraceous species in the central Chilean Andes (Primack 1985). Stigmas remain receptive for at least 7 days [**see Supporting Information—Figure S1**]. Anthers contain large pollen grains which are easily distinguished on stigmas with a hand-lens (Ladd and Arroyo 2009). Flowers are pollinated by *Megachile saulcyi* (Megachilidae) (Ladd and Arroyo 2009). This bee uses *Adesmia coronilloides* and *Phacelia secunda* as its main nectar sources.

The present study took place in the 2014–15 austral summer on three sites located between 2350 and 3075 m a.s.l. (see Table 1 for site details). Potential flower longevity measured over the same summer on these study sites can be found in Table 1. Earlier work on *R. montanum* in the same general area failed to detect pollen limitation (Ladd and Arroyo 2009). However, in that study, the sample sizes were severely reduced by browsing cattle and elevational variation in pollen limitation was not addressed.

**Table 1.** Potential flower longevity (measured in pollinator-excluded flowers), realized flower longevity (measured in open-pollinated flowers) and the ratio of realized to potential flower longevity in *Rhodolirium montanum* at three elevations in the high central Chilean Andes (33° S) along with site characteristics. Different letters for realized flower longevity indicate significant differences. Potential flower longevity, measured on the same sites and for the same summer season, is from Pacheco *et al.* (2016); differences between potential longevity for all pairs of sites were significant.

Site	Altitude (m a.s.l.)	Vegetation characteristics	Mean potential flower longevity in days	Realized flower longevity in days, mean ± SE	Ratio realized: potential flower longevity
LOW	2350	Subalpine belt immediately above the treeline	5.7	3.60^a^ ± 0.146 (*N* = 53)	0.63
MID	2650	Transition between the subalpine and high alpine belts	6.3	4.75^b^ ± 0.170, (*N* = 57)	0.75
HIGH	3075	High alpine belt	7.8	5.20^b^ ± 0.208, (*N* = 55)	0.67

#### Flower visitation.

We quantified flower visitation rates across the whole flowering season on each site. Individual sites were visited at 3-day intervals. Each day two (exceptionally one) 2 m × 2 m plots were observed by separate observers, each for two 20 min periods per hour from 0900 to 1800 h irrespective of weather conditions. Plots on a site were relocated on new observation days to cover local variation. Total observation days per site were five (HIGH, MID) and six (LOW). We recorded the total number of open flowers, all visits to those flowers, and visitor identity. Total 20 min observations were LOW = 215; MID = 175; and HIGH = 162. To investigate the relationship between flower visitation rates and temperature, we obtained air temperature from shaded Hobos (Onset Computer Corp., Cape Cod, MA) set up at 15 cm.a.g.l to capture temperature conditions around flowers.

#### Pollen limitation.

We measured pollen limitation with the standard supplemental pollination method. Pairs of genets were haphazardly selected with one fresh flower marked per genet. The flowers were randomly assigned to a control and a supplemental pollination treatment. Supplemented flowers were pollinated with a mixture of fresh pollen obtained from a minimum of three distant plants. Total numbers of pollination days—pairs of flowers per site were 9—165 (LOW); 10—174 (MID); and 9—114 (HIGH). Final sample sizes discarding losses were LOW: control = 156, supplemented = 147; MID: control = 109, supplemented = 128; and HIGH: control = 96, supplemented = 92. The single-flowered bulbs of *R. montanum* are essentially independent physiological units. Consequently, our study qualifies as a whole-plant pollen limitation study.

Total ovules and good seed were counted for all retrieved fruits. With the aim of comparing abortion levels in the control and pollen-supplemented flowers, for large sets of fruits we further separated the ovules that had not matured into good seed into (i) unfertilized ovules and (ii) abortions. Small aborted embryos could be seen in intermediate-sized fertilized ovules. For the detection of early abortions we relied on ovule size and shape.

#### Pollen deposition on stigmas and realized flower longevity.

To detect when pollen first appeared on stigmas in the flower lifespan and pollen dosage intensities we examined flowers for conspecific pollen deposition. Observations were made on 60 haphazardly selected genets per site in the middle of the flowering season over the same dates. One flower bud was selected per genet. As the flowers opened, we established the first day upon which they received pollen by checking their stigmas with a ×40 bright hand lens early in the morning before bees became active. Final sample sizes, discarding damaged flowers, were LOW: *N* = 55; MID: *N* = 57; and HIGH: *N* = 55. Because the main aim was to detect if stigmatic pollen deposition is delayed at higher elevations, individual stigmas were no longer observed once they had received some pollen. To compare pollen dosage levels at different elevations, we visually estimated the amount of pollen deposited on individual stigmas according to the following coverage categories: 1 = no conspecific pollen received, 2 = up to ¼; 3 > ¼–½; 4 ≥ ½–¾; 5 ≥ ¾. Pollen loads correspond to visits made over a single day. Thus, the amount of pollen recorded provides an estimate of pollen dosage intensity.

To determine whether pollen deposition ceased progressively later in the flower lifespan with increasing elevation we determined how long open-pollinated flowers remained open. Realized flower longevity was obtained from anthesis and floral senescence dates recorded in the field on the same three sets of 60 flowers. As noted earlier, potential flower longevity in the year of study was reduced at low elevation due to soil drying. Realized flower longevity could also be affected at low elevation if soil drying hastens post-pollination senescence. We therefore also calculated the ratio of realized to potential flower longevity for each site (Table 1), which under flower longevity compensation is expected to increase with elevation.

The timing of floral senescence following pollination varies among species (O’Neill 1997; van Doorn 1997). We carried out complementary hand-pollination experiments (on HIGH and LOW) to determine whether realized flower longevity provides a precise proxy for when stigmatic pollen deposition terminates in a flower. Rapid closure of flowers after ample hand-pollination would indicate flower senescence is strongly pollination-triggered and a high level of precision. On each site, 70 pollinator-excluded flowers (one per genet) were assigned to seven equal-sized groups for pollination on successive days after anthesis. We pollinated stigmas with a mixture of fresh pollen obtained from distant genets. We monitored the pollinator-excluded stigma cohorts until floral senescence. This allowed us to obtain the number of additional days flowers remained open after they had been pollinated.

#### Data analysis.

Visitation rates were expressed as the total number of visits per 20 min divided by the total number of flowers observed. To depict daily variation in visitation rates, we calculated hourly median visitation rates and temperatures 0900–1800 h. We averaged the two simultaneous 20 min observations. To detect site differences in flower visitation rates and their relationship with temperature we calculated mean overall daily visitation rates and mean daily temperatures. Daily visitation rates were analysed with a negative binomial model with site as a categorical variable using the MASS package (Venables and Ripley 2002). To investigate whether temperature explains variation in visitation rates among sites, we developed a second model in which temperature was included as a covariate.

For pollen limitation, differences in plant density among sites and variation in available flowers over the season made it impossible to maintain a standard distance between control and supplemental flowers as was originally planned. We, therefore, analysed our data in a non-paired design, using the seed/ovule ratio. Pollen limitation was analysed with a Zero-Inflated Negative Binomial (ZINB) model (Hilbe 2014). This model is appropriate when there are large numbers of zeros due to lack of pollination or total fruit abortion. Treatment (control, supplemental pollination) and site (HIGH, MID, LOW) were considered as categorical variables. Data were analysed with the *pscl package* (Jackman 2015) which uses the Zeileis *et al.* (2008)*zeroinfl* function. We also compared the proportions of flowers setting fruit in the control and pollen-supplemented flowers with the *Z*-test for proportions. Abortion levels for control versus pollen-supplemented flowers were analysed with the Mann–Whitney *U* test.

To visualize differences among sites regarding when stigmatic pollen deposition commences in the flower lifespan we calculated the proportions of monitored flowers that had failed to receive conspecific pollen on increasing numbers of days after anthesis. To compare pollen dosage intensities, the frequencies of stigmas in the different pollen coverage categories were analysed with Pearson’s χ^2^ test. Realized flower longevity was analysed with the Kruskall–Wallis test. We used Dunn’s test for multiple comparisons with the Bonferroni correction for *a posteriori* comparisons among pairs of sites. The number of days flowers remained open after pollination in relation to flower age at the time of hand-pollination was depicted graphically.

Models and statistical tests were performed in R version 3.3.2. (R Core Team 2016).

## Results

### Flower visitation rates

We registered a total of 4377 flower visits across the three sites. *Megachile saulcyi* was by far the most important pollinator (96.16 % of visits). A second *Megachile* species (*M. semirufa*), found almost exclusively on HIGH, contributed a mere 0.32 % of the visits. Small percentages of visits were registered for other hymenopterans (0.16 %), dipterans (3.18 %), coleopterans (0.11 %) and lepidopterans (0.07 %).

**Figure 2.**
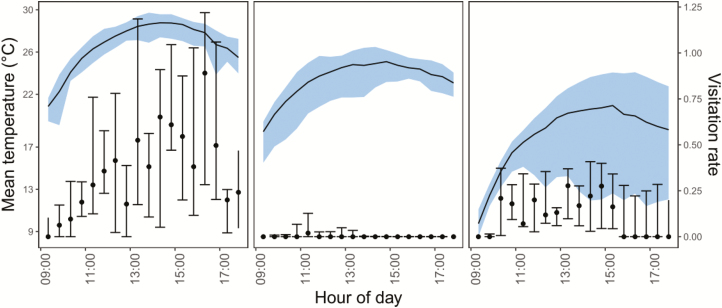
Mean median daily flower visitation rate (number of visits per flower per 20 min) (points) and mean daily temperature at 15 cm a.g.l (continuous line) according to hour of the day on three sites representing *Rhodolirium montanum*’s elevational range in the central Chilean Andes. See Table 1 for site details. Vertical bars give the first and third quartile for visitation rates. Blue shading portrays the difference between maximum and minimum temperatures recorded hourly over the day. See text for sample sizes.

As expected, temperature decreased notably with elevation (Fig. 2). Mean hourly temperature around flowers rose to over 25 °C after midday on LOW (Fig. 2), but never beyond 20 °C on HIGH. Visitation tended to commence later in the morning and tapered off earlier in the afternoon as elevation increased. Moreover, temperature for any given hour of the day was more variable with increasing elevation.

**Figure 3.**
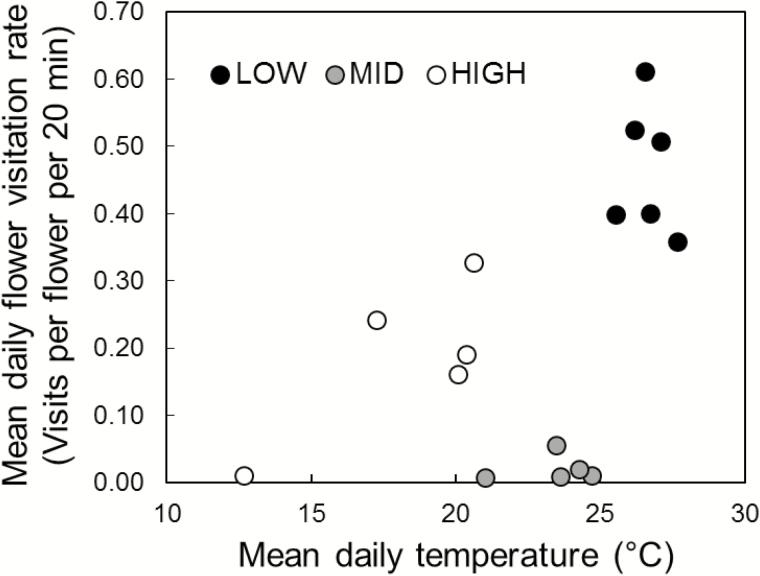
Mean flower visitation rates compared with mean temperature on each day of pollinator observation on three sites representing the elevational range of *Rhodolirium montanum* in the high central Chilean Andes. See Table 1 for site details.

Mean visitation rates and temperatures for sites are shown in Fig. 3. Site had a significant effect on mean daily visitation rate (log likelihood ratio = 122.19, *P* < 0.001), with all differences between pairs of sites being significant: HIGH versus LOW (*Z* = −3.999, *P* < 0.0005); HIGH versus MID (*Z* = 8.033, *P* < 0.0001); MID versus LOW (*Z* = −6.960, *P* < 0.0001). The mean daily visitation rate on LOW (0.47 visits flower^−1^ 20 min^−1^) was 2.5 times greater than on HIGH (0.19 visits flower^−1^ 20 min^−1^), signifying that visitation rates on HIGH declined by 59.6 %. These visitation rates translate to 12.7 and 5.1 visits per flower per day (including revisits), respectively (calculated from the mean visitation rate and a 9-h pollination day). The mean daily visitation rate on MID (0.02 visits flower^−1^ 20 min^−1^, equivalent to 0.54 visits per flower per day), contrary to prediction given its intermediate position on *R. montanum*’s elevational range, was exceedingly low. Comparatively speaking, flower visitation rates on LOW and HIGH were 23.5 and 9.4 times higher than on MID, respectively.

Upon including temperature as a covariate in the model, the difference in visitation rates for HIGH and LOW disappeared (Contrast of marginal means = 0.482 [visits flower^−1^ 20 min^−1^]; *Z* = 1.146, *P* = 0.4860) suggesting, that, as expected, temperature was probably responsible for the difference in visitation rates among these sites. However, temperature could not explain the MID visitation rates (LOW vs MID: Contrast of marginal means = 3.311 [visits flowers^−1^ 20 min^−1^]; *Z* = −5.406, P < 0.0001; HIGH vs MID: Contrast of marginal means = 3.792 [visits flowers^−1^ 20 min^−1^]; *Z* = 6.000, P < 0.0001). This result indicated that some other factor was responsible for the unusually low visitation rates on MID.

### Pollen limitation

**Figure 4.**
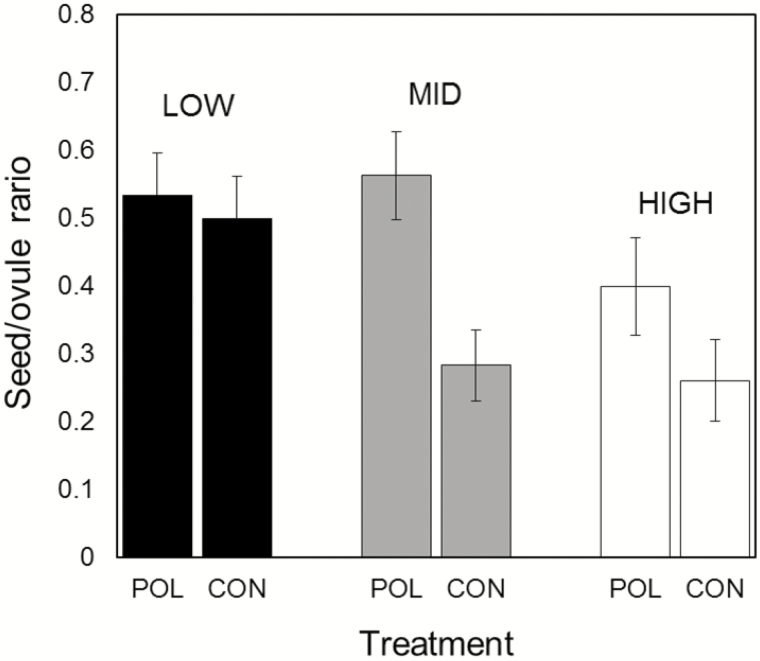
Seed/ovule ratio in control (CON) and supplementally pollinated flowers (POL) on three sites representing *Rhodolirium montanum*’s elevational range in the Andes of central Chile. Vertical bars = confidence interval of the mean.

Supplemental pollination failed to increase the seed/ovule ratio on LOW, revealing the absence of pollen limitation on the lower part of the elevational range (Fig. 4). On HIGH, supplemental pollination increased seed set by 53 %, while on MID this figure rose to 99 %, indicating considerable pollen limitation on the two higher sites, and especially on MID. In agreement with these results, the proportion of pollen-supplemented flowers forming fruits did not exceed that of control flowers on LOW (*Z* = 0.3804, *P* = 0.704), whereas differences were significant on MID (*Z* = 3.702, *P* < 0.001) and HIGH (*Z* = 2.249, *P* < 0.05). These last results suggest that failures in pollination or in pollen tubes reaching the ovary contributed to pollen limitation on these two sites. In general, we observed high levels of embryo and seed abortion both in the control and pollen-supplemented flowers, increasing with elevation (Table 2). Abortion levels on LOW were not significantly different between control and supplemented flowers. With increasing elevation, however, there was a distinct tendency for more abortion in the control flowers than in the supplemented flowers (Table 2). Higher abortion levels in the controls occurred precisely where *R. montanum* forms large clones.

**Table 2.** Early embryo and seed abortion levels in fruits for control and pollen-supplemented flowers of *Rhodolirium montanum* on three high Andean sites. See Table 1 for elevations of sites.

Site	Control, mean ± SE	Pollen supplemented, mean ± SE	Mann–Whitney *U* test
LOW	43.06 ± 4.567 (*N* = 54)	37.08 ± 5.189 (*N* = 35)	*P* = 0.352
MID	60.12 ± 5.136 (*N* = 38)	38.32 ± 4.915 (*N* = 48)	*P* = 0.004
HIGH	59.09 ± 3.789 (*N* = 62)	49.59 ± 3.738 (*N* = 73)	*P* = 0.060

### Pollen deposition on stigmas and realized flower longevity

**Figure 5.**
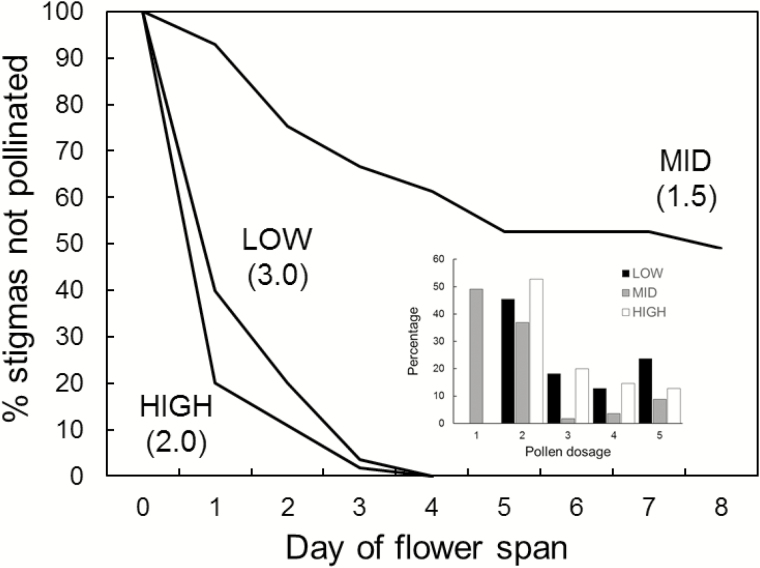
Percentages of monitored open-pollinated flowers on three sites representing *Rhodolirium montanum*’s elevational range that had not received conspecific pollen on their stigmas on increasing numbers of days as of anthesis together with corresponding median pollen dosage intensity levels (numbers below site names) for each site. For flower age, 1 = day of anthesis, and successively; 0 = flower still not open. Inset gives the frequency distribution of pollen dosage levels. See methods for explanation of pollen dosage intensity levels.

Proportions of stigmas that failed to receive conspecific pollen with increasing time after anthesis (Day 1) and pollen dosage intensities on stigmas are shown in Fig. 5. Pollen dosage intensity was heterogeneous among sites (χ^2^ = 8, *P* < 0.0001). On all sites, receipt of initial pollen on stigmas occurred over several days of the flower lifespan. At both the HIGH and LOW sites, high proportions of the stigmas began receiving pollen on the first day of anthesis and within 4 days all flowers had received a first dose of pollen. Although HIGH-site flowers were visited relatively earlier, pollen dosage intensities on stigmas were lower (Fig. 5) signifying fewer visits on a given day. The pattern of pollen deposition for MID flowers was strikingly different (Fig. 5). Here flowers began to receive pollen much more gradually to the extent that fully 49.1 % of the flowers failed to receive pollen by the time of closure. Not surprisingly, reflecting the very low visitation rates on this site, pollen dosage intensity on MID stigmas was lowest.

Site had a significant effect on realized flower longevity (Kruskal–Wallis χ^2^= 39.471, df = 2, *P* < 0.0001) (Table 1). Realized flower longevity on HIGH was significantly greater than on LOW (*P* < 0.0001), indicating that, although HIGH flowers received less pollen over a day of visitation, on average they would have continued receiving pollen until later in their flower lifespans. Nevertheless, in view of the persistent pollen limitation at this elevation, the total amount of pollen received over the flower lifespan was evidently insufficient to achieve the maximum physiological seed set as occurred at LOW. Realized flower longevity was significantly shorter at LOW than at MID (*P* < 0.0001), but this was not the case for MID versus HIGH (*P* = 0.3700). In agreement with the much lower MID visitation rates, the ratio of realized to potential flower longevity for this site was higher than for both LOW and HIGH (Table 1). LOW exhibited the lowest ratio of all sites.

**Figure 6.**
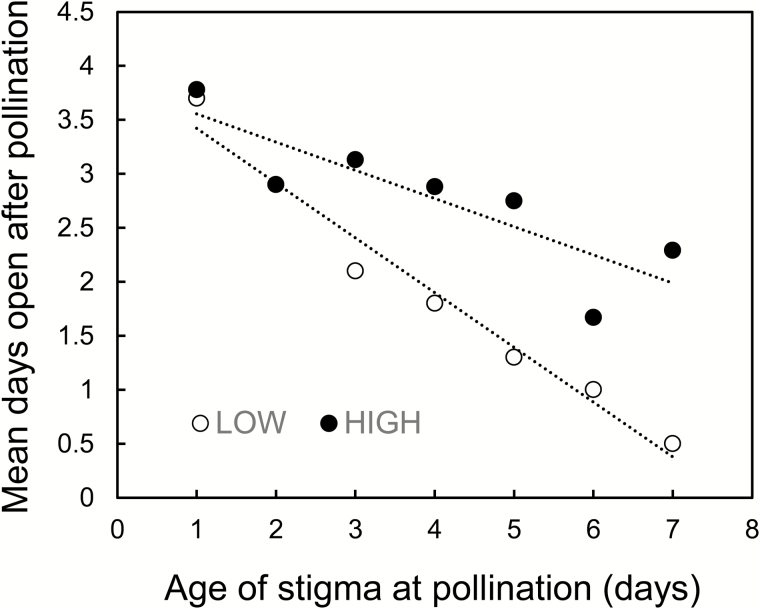
Mean number of days flowers continued to remain open following hand-pollination on the lower (LOW) and upper (HIGH) extremes on the elevational range of *Rhodolirium montanum*. The dotted lines show the general tendencies for the two elevations.

Results of the hand-pollination experiment designed to ascertain how quickly flowers close after an initial pollination event are shown in Fig. 6. Flowers pollinated on the day of anthesis (Day 1) remained open for means of 3.7 (LOW) and 3.8 (HIGH) additional days. These results (considering copious pollen was applied to stigmas), indicate a fairly mild effect of pollination on floral senescence and suggest initial deposition of pollen on stigmas would not have precluded receipt of additional pollen later. There was a tendency for flowers to remain open for fewer additional days after pollination when they were pollinated later in their life-spans. This trend was more notable on LOW. Overall, flower longevity (mean ± 1 SE) for the full sets of hand-pollinated flowers (LOW: 5.64 ± 0.158; HIGH: 6.67 ± 0.236) was as expected, shorter than potential flower longevity (cf. Table 1).

## Discussion

Here, we set out to determine whether pollen limitation intensity remains invariant over the elevational range of a high Andean outcrossing amaryllid, in spite of an expected decrease in flower visitation rates due to rapidly declining air temperatures above the treeline as shown earlier at a community level on our study sites (Arroyo *et al.* 1985). We hypothesized that similar levels of pollen limitation could be maintained over the elevational range on account of our focal species possession of intrinsically long-lived flowers. According to this hypothesis, we expected pollen deposition on stigmas at high elevation to commence and terminate later in the flower lifespan and/or to occur in smaller daily doses. The weight of the evidence points to partial flower longevity compensation of lower visitation rates at high elevation in *R. montanum*. However, such compensation does not translate into a commensurate reduction in overall pollen limitation due to additional qualitative pollen limitation at higher elevations.

On the lower end of the range we found high visitation rates and a complete absence of pollen limitation. Relative to the lowest site, our highest site experienced a 60 % decrease in visitation rates which was associated with substantial pollen limitation. Visitation rates were exceedingly low, and pollen limitation was very high in the middle of the range. That site turned out to be anomalous due to heavy grazing on the key nectar plant (*Adesmia coronilloides*) of *R. montanum*’s main pollinator which significantly lowered bee abundance.

With total flower longevity compensation of the lower visitation rates on the upper part of the range, we expected supplemental pollination to produce no increase in seed set over the controls. This was evidently not the case. At the other extreme, under no flower-longevity compensation, supplemental pollination should have led to a 60 % increase in seed set over that in the controls. Supplemental pollination on the high site boosted seed set by 53 %. This implies a negligible 7 % reduction in pollination limitation attributable to the flower longevity effect. This estimate assumes that visitation levels on the lower part of *R. montanum*’s elevational range are not higher than required for maximum seed set. At present, we have no way of telling whether this assumption is valid. The 7 % estimate, however, assumes flower visitation rates to be the only factor driving pollen limitation in *R. montanum*.

Our results suggest that some of the pollination limitation at high elevations may reflect qualitative (as opposed to more typical quantitative) pollen limitation caused by the deposition of genetically low-quality pollen on stigmas (cf. Ramsey and Vaughton 2000; Thomson 2001; Finer and Morgan 2003; Aizen and Harder 2007). We found a general tendency for increased levels of abortion with increasing elevation in *R. montanum*, which is common in alpine species (Körner 2003). Of far greater relevance in the present context are the higher levels of abortion in control flowers compared to pollen-supplemented flowers on the two highest sites where *R. montanum* is strongly clonal. Such higher levels of abortion are best explained by geitonogamous selfing on a partially self-compatible plant (cf. Cosacov *et al.* 2008; Hu *et al.* 2015). Pollen-supplemented flowers would have been less susceptible to receiving genetically low-quality pollen because the pollen used came from distant and likely genetically distinct plants, and hence the lower abortion levels. Wirth *et al.* (2010) provided good genetic evidence for geitonogamous pollination in the alpine cushion plant *Eritrichium nanum*, a species characterized by large numbers of simultaneously blooming flowers.

To arrive at the real level of longevity compensation for low visitation rates at high elevations, qualitative pollen limitation must be factored out. When this is done by increasing seed set to equal abortion levels in the control and supplemented flowers (obtained by reducing abortion levels in the controls by 9.5 %), the increase in seed set with supplemental pollination on the upper part of the elevational range decreases from 53 % to 37 %. Accordingly, the estimated buffering effect of long-lived flowers on the lower visitation rates rises to 23 %. Based on these calculations, *R. montanum*’s long-lived flowers are seen to partially compensate the 60 % reduction in lower visitation rates at high elevations. However, this not insignificant flower longevity benefit is not immediately obvious because it is strongly masked by apparent qualitative pollen limitation.

Partial flower longevity compensation of quantitative pollen limitation was also supported by patterns of stigmatic pollen deposition patterns and realized flower longevity. Although pollen deposition tended to begin early on stigmas on the upper end of the range, pollen dosage intensity here was lower. This is not surprising in view of the much lower number of visits received by individual flowers over a day at high elevations, as per the flower visitation rates. High-elevation open-pollinated flowers (realized flower longevity) did indeed remain open for longer than their low elevation counterparts, as was expected under flower longevity compensation. This is consistent with pollination deposition occurring over a larger part of the flower lifespan at high elevation. Flowers that remain open after an initial pollination event, as occurs in *R. montanum,* allow multiple pollinator visits, thereby increasing the number of pollen donors and obtaining the resultant benefits of pollen competition (Kalla and Ashman 2002). Nevertheless, although later pollen deposition would have been possible at high elevation, given the dynamics of post-pollination floral senesence in *R. montanum*, realized flower longevity cannot provide a precise answer regarding when pollen deposition ceased in the flower lifespan in the high elevation flowers, nor for that matter in the low elevation flowers.

Qualitative pollen limitation driven by extensive clonal growth at high elevations in *R. montanum* is one of the more interesting findings coming out of our study. Clonal growth facilitates persistence under suboptimal environmental conditions (Kudoh *et al.* 1999; Erikson and Ehrlén 2001). It simultaneously promotes increased attraction of pollinators by providing larger floral displays (Harder and Barrett 1996; Vallejo-Marín *et al.* 2010). The last effect should be advantageous for securing mates when visitation rates are low as occurs at high elevation. Such advantages contrast with the disadvantages coming from lower adaptability to changing environmental conditions, and, as seen in *R. montanum*, a propensity for geitonogamous selfing in species with some self-compatibility leading to seed discounting. Stronger clonal growth at high elevations in *R. montanum* would tend to counteract lower fitness due to residual pollen limitation driven by low visitation rates not compensated by flower longevity. However, clonal growth is also expected when environmental conditions are limiting for germination and establishment, as a lack of young plants on the upper part of *R. montanum*’s range seems to indicate. These factors would reinforce each other and be conducive to the selection for extensive clonal growth at higher elevations. Overall, in *R. montanum*, the long-term fitness gains of clonal growth in the harsh alpine environment appear to outweigh the seasonal fitness losses coming from quantitative and qualitative pollen limitation combined, to the extent that both sources of pollen limitation are tolerated. Many alpine species are clonal (Keller and Vittoz 2015) or form flat cushions (Aubert *et al.* 2014) that bear large numbers of synchronously open flowers and thus are susceptible to geitonogamous pollen transfer. Combinations of quantitative and qualitative pollen limitation thus are likely to be common in alpine species and come under distinct guises (cf. Kasagi and Kudo 1993).

The mean visitation rate recorded on our lower site was about six times the community average reported in the same general area several decades ago (Arroyo *et al.* 1985). The central Chilean Andes warmed by about 0.28 °C per decade from 1979 to 2006 (Falvey and Garreaud 2009) signifying close to a 1 °C increase in temperature. Are current visitation rates and levels of pollen limitation in *R. montanum* representative of historical levels before the onset of global warming? According to the effect size coming out of the analysis of visitation rates in relation to temperature, a 1 °C temperature increase should have boosted flower visitation rates in *R. montanum* by 19 %. Current levels of pollen limitation thus could be lower than historical levels. Nevertheless, under cooler conditions, in species with long-lived flowers, pollen deposition could have been spread out over a larger portion of the flower lifespan and thereby partially compensate the historically lower visitation rates.

## Conclusions

The much lower visitation rates in the upper part of *R. montanum*’s elevational range are only partially offset by its long-lived flowers. The benefits of flower longevity compensation at high elevation are largely obscured by qualitative pollen limitation attributable to the transference of genetically low-quality pollen on large clones which increases the level of pollen limitation over that driven by lower visitation rates. The long-term fitness benefits of clonal growth in the harsh alpine environment appear to outweigh the seasonal fitness losses associated with quantitative pollen limitation to the extent that additional qualitative pollen limitation is tolerated. Overall, the elevational trend in pollen limitation in *R. montanum* is not analogous to that revealed for alpine versus lowland species as per the García-Camacho and Totland (2009) meta-analysis. Pollination limitation measured appropriately across the entire flowering season and elevational ranges in other alpine species might show a similar departure. Finally, current levels of pollen limitation in alpine and other species might not always represent historical levels before global warming. The latter has implications for understanding traits that avoid pollen limitation, given that what we see today could have evolved under different environmental conditions.

## Sources of Funding

This work was supported by grants Fondecyt 1140541 to M.T.K.A. and ICM-MINECON P05-002 and PBF-23 to the Instituto de Ecología y Biodiversidad. D.A.P was supported by a CONICYT-PCHA/Magíster Nacional/2013—22131579 Fellowship.

## Contributions by the Authors

M.T.K.A., D.A.P. and L.S.D. designed the research; M.T.K.A., D.A.P and L.S.D. performed the field work; D.A.P. and M.T.K.A. analysed the data; M.T.K.A., D.A.P. and L.S.D. wrote the paper.

## Conflicts of Interest

None declared.

## Supporting Information

The following additional information is available in the online version of this article—

**Figure S1.** Duration of stigma receptivity in *Rhodolirium montanum* based on levels of pollen germination on stigmas of different ages (days).

## Supplementary Material

Supporting Material
