# Supplementary material for: Functional role of long-lived flowers in preventing pollen limitation in a high elevation outcrossing species
Source: AoB Plants. 2017 Oct 21;9(6):plx050. doi: 10.1093/aobpla/plx050 (PMC5716155; doi:10.1093/aobpla/plx050)
Supplement: Supporting Material [file plx050_suppl_supporting_material.docx]

**Supporting Information**

**Figure S1.** Duration of stigma receptivity in *Rhodolirium montanum* based on levels of pollen germination on stigmas of different ages (days) following hand pollination on LOW (A) and HIGH (B)*.*


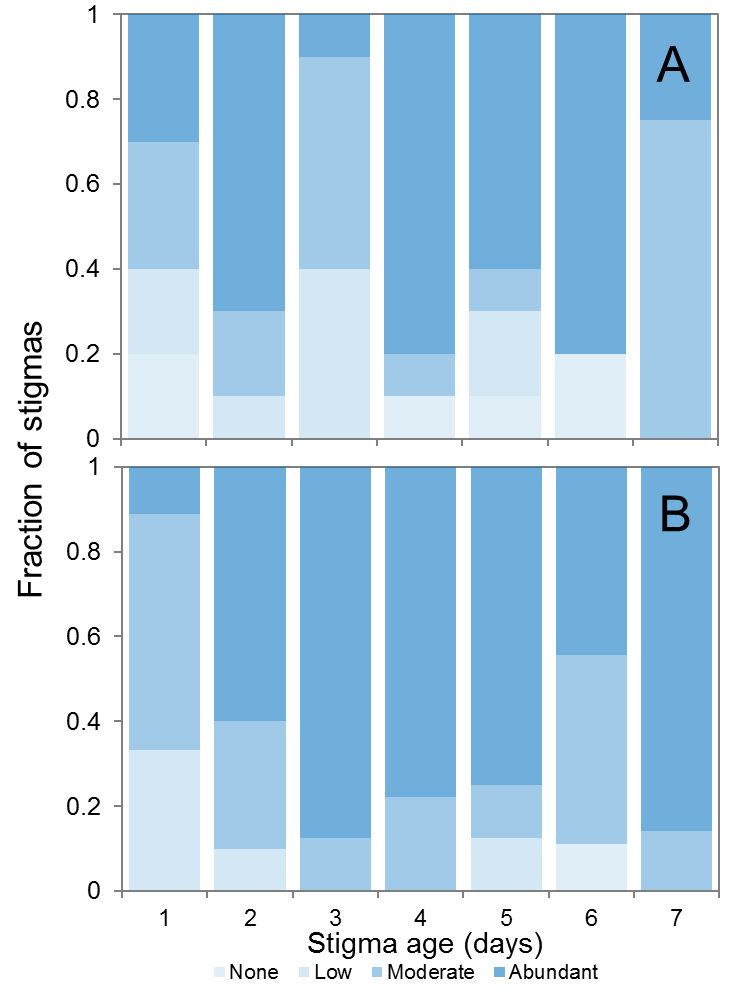


Levels of pollen germination were obtained from the seven cohorts of stigmas pollinated at different ages, used to study post-pollination flower senesence (see Materials and Methods). Final sample sizes after were: LOW = 59; HIGH = 60. The available stigmas and part of the styles were excised minimally two days later and preserved in 70% alcohol: acetic acid (3:1). Material was stained in a solution of 0.01% aniline blue dissolved in 0.05 M K_2_HPO_4_ for two hours in the dark, squashed in glycerine, and imaged under fluorescence microscopy. Pollen germination was visually ranked at the stigma/style junction according to the following approximate pollen germination categories. None - no pollen grains with visible pollen tubes; Low - up to around 1/3 with visible tubes; Moderate - > 1/3-2/3 with visible tubes; Abundant - > 2/3 with visible tubes. Neither stigma age $X$^2^ = 0.87, d.f. = 2, P = 0.6473) nor site had an effect on the proportion of stigmas with germinating pollen ($X$^2^ = 1.67, d.f. = 2, P = 0.4334) indicating that stigmas of *R. montanum* remain receptive for at least seven days.
